# Central Aortic Cannulation for Total Coronary Revascularization via Anterior Thoracotomy: A Single-Center Initial Experience

**DOI:** 10.3390/jcdd13030123

**Published:** 2026-03-07

**Authors:** Tuna Demirkıran, Işıl Taşöz Özdaş, Gizem Işık Ökten, Furkan Burak Akyol, Tayfun Özdem, Yiğit Tokgöz, Hüma Kekeçdil, Murat Kadan, Kubilay Karabacak

**Affiliations:** 1 Department of Cardiovascular Surgery, Gulhane Training and Research Hospital, Ministry of Health, Ankara 06010, Türkiye; isil.tasoz@sbu.edu.tr (I.T.Ö.); furkanburak.akyol@sbu.edu.tr (F.B.A.); tayfun.ozdem@sbu.edu.tr (T.Ö.); 2 Department of Cardiovascular Surgery, Gulhane Training and Research Hospital, University of Health Sciences, Ankara 06010, Türkiye; gizem.isik1@sbu.edu.tr (G.I.Ö.); yigit.tokgoz@sbu.edu.tr (Y.T.); huma.kekecdil@sbu.edu.tr (H.K.); kubilay.karabacak@sbu.edu.tr (K.K.); 3 Department of Cardiovascular Surgery, Memorial Ankara Hospital, Ankara 06510, Türkiye; murat.kadan@memorial.com.tr

**Keywords:** central aortic cannulation, TCRAT, total coronary revascularization, antegrade perfusion, cardiopulmonary bypass

## Abstract

Objective: We aimed to evaluate the feasibility, safety, and technical challenges of central aortic cannulation for total coronary revascularization via left anterior thoracotomy (TCRAT). Methods: A retrospective, single-center observational study was conducted on the first 29 TCRAT cases performed with central aortic cannulation. The primary outcomes included operative mortality, stroke, conversion to sternotomy, major aortic bleeding, and dissection; the secondary outcomes included delirium, reoperation, infection, ICU stay, and hospitalization. The descriptive statistics were reported as means ± SD or median (interquartile range [IQR]). Results: The mean age of the patients was 57.2 ± 9.8 years, with 72% of these being male. The most frequent comorbidities observed in the study population were hypertension (62%), diabetes (52%), and peripheral artery disease (28%). The mean cross-clamp time was found to be 63 ± 27 min, and the mean CPB time was 118.6 ± 41.6 min. The occurrence of stroke, aortic dissection, major bleeding, and sternotomy conversions was not observed. One patient died from severe pneumonia on the ninth post-operative day. The mean ICU stay was 1.2 ± 0.4 days, and the mean hospital stay was 5.3 ± 1.1 days. Conclusions: Central aortic cannulation appears to be a safe and feasible procedure for TCRAT, providing physiological antegrade flow and eliminating the complications associated with peripheral cannulation. The preliminary findings suggest that central arterial cannulation may be a safe and practical alternative for the TCRAT technique, but prospective comparative studies are required to confirm its benefits over the femoral and axillary approaches.

## 1. Introduction

Technological advances in percutaneous coronary interventions, supported by industry, combined with the successful application of laparoscopic minimally invasive techniques in other surgical specialties, have encouraged the development of less invasive approaches in cardiac surgery. Since the 1990s, significant progress has been made in minimally invasive, endoscopic, and robot-assisted techniques for coronary artery bypass surgery [1,2,3]. However, due to technical and economic challenges, as well as issues such as inadequate revascularization, no procedure has yet replaced traditional surgery with sternotomy or become widely adopted in clinical practice for total coronary revascularization. Minimally invasive off-pump coronary artery bypass surgery offers significant advantages. However, surgery on an arrested heart provides the surgeon with a motionless, bloodless field, allowing for more precise, easier anastomoses. On the other hand, sternum-sparing approaches have become more popular over the last decade. The total coronary revascularization with anterior thoracotomy (TCRAT) technique, as defined by Babliak et al. in 2019, has proven to be a safe and routinely feasible method for complete coronary revascularization [4]. This technique involves inducing cardioplegic arrest of the heart under cardiopulmonary bypass and cross-clamp. This technique is similar to traditional surgical principles and can be routinely used in most patients [5]. TCRAT has been successfully conducted at our institution since February 2021.

In minimally invasive cardiac surgery, cannulation for cardiopulmonary bypass has a significant impact on surgical success and clinical outcomes. The development of minimally invasive techniques has led to the emergence of new cannulation strategies. In the TCRAT, peripheral arterial cannulation is usually performed, most often through the femoral artery [4]. However, femoral arterial cannulation can cause retrograde aortic dissection, neurological complications related to retrograde perfusion, and vascular complications on the cannulation site, distal limb ischemia, and wound complications [6,7]. An alternative to femoral artery cannulation in TCRAT is axillary arterial cannulation, as used in complex aortic surgeries. However, this technique also has many morbidities, such as peripheral complications, malperfusion, or brachial plexus pathologies [8].

Central arterial cannulation is another option for minimally invasive cardiac surgery [9,10]. The primary benefits of central arterial cannulation include a low risk of plaque embolization due to physiological (antegrade) flow and the avoidance of complications associated with peripheral arterial cannulation. However, due to technical challenges, it has been performed in only a few centers worldwide and solely for valve surgery. In our institution, central arterial cannulation has been increasingly used in valve surgeries over the past 10 years.

This study presents initial findings from the first 29 patients who underwent TCRAT with central arterial and percutaneous venous cannulation for cardiopulmonary bypass at our clinic. The study aims to evaluate the feasibility, safety, and technical challenges of the TCRAT procedure using central arterial cannulation.

## 2. Materials and Methods

### 2.1. Ethics Approval and Informed Consent

The institutional ethics committee approved the study protocol (Gulhane Health Practice and Research Center Scientific Research Evaluation Committee, Date: 8 January 2025, No: 1). The study was conducted in accordance with the principles of the Declaration of Helsinki. Because of the study’s retrospective design and the use of anonymized patient data, the ethics committee waived the requirement for written informed consent.

### 2.2. Study Design and Patient Population

The present study is a retrospective, single-center observational study. The TCRAT procedure has been performed at our clinic since February 2021. The study period spanned from February 2021 to December 2024. During this timeframe, 138 patients underwent TCRAT. Of these, 29 underwent TCRAT with central aortic and percutaneous femoral venous cannulation. All patients who used this cannulation strategy during the study period were included, representing our initial experience with it for TCRAT. All data were recorded prospectively and analyzed retrospectively. Over the past ten years, there has been a significant increase in the use of central arterial cannulation for minimally invasive procedures at our clinic, particularly for valve surgeries performed through right thoracotomy. Central arterial cannulation was preferred in selected TCRAT cases (e.g., patients with extensive descending aortic atherosclerosis, a risk of femoral access site complications due to morbid obesity, or peripheral arterial disease), where the goal was to avoid complications associated with peripheral arterial cannulation. Preoperative computed tomography (CT) angiography was performed in patients with a significant atherosclerotic burden or known peripheral arterial disease. For patients unable or unwilling to undergo CT angiography, preoperative ultrasound of the iliofemoral axis was performed, along with intraoperative transoesophageal echocardiography (TEE) of the aortic arch and thoracic aorta. Investigations found that atheromas classified as Grade IV or V that protruded into the lumen or were mobile were contraindications to femoral cannulation and retrograde perfusion. Therefore, central cannulation was prioritized. The primary indication for central arterial cannulation has been in patients with extensive descending aorta atherosclerosis (grade IV or V), those at high risk of femoral access site complications due to morbid obesity (Femoral region, according to clinical assessment), or those diagnosed with peripheral arterial disease.

### 2.3. Surgical Technique

Our previous publication provided a comprehensive description of the surgical technique for TCRAT performed at our institution [6].

**Figure 1:**
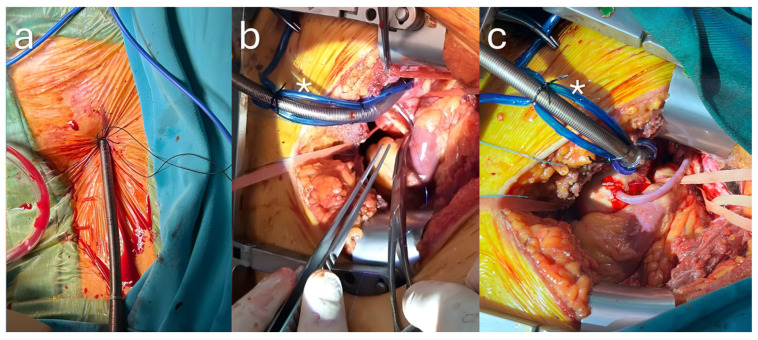
(**a**) Percutaneous peripheral bicaval venous cannulation. (**b**) Proximal anastomosis performed with a side clamp on the ascending aorta. (**c**) Final appearance of the completed proximal anastomosis. The asterisk (*) in panels (**b**,**c**) marks the central aortic cannula.

When central arterial cannulation was planned, thoracotomy was routinely performed through a 7–8 cm skin incision at the third intercostal space. A specially designed retractor (Delacroix-Chevalier, Paris, France) was used during the harvesting of the Left Internal Thoracic Artery (LITA) graft. To optimize graft length, the LITA was not divided initially; instead, the proximal segment was harvested first, the retractor was reversed, and harvesting continued toward the distal segment. After pericardiotomy, the space between the aorta and pulmonary artery was dissected using an electrocautery device, and the ascending aorta was encircled with a 10 mm vascular tape. Two pledgetted-purse-string sutures were applied to the distal anteromedial surface of the ascending aorta, ensuring sufficient working space for the proximal anastomosis. The adventitia is carefully excised in the cannulation area. The next step involves the administration of pharmacologically induced hypotension, defined as a systolic blood pressure of less than 90 mmHg. After heparinisation, ultrasonography-guided percutaneous peripheral bicaval venous cannulation via a Bio-Medicus™ NextGen Femoral Venous Cannula (Medtronic Inc., Minneapolis, MN, USA) is performed under TEE guidance before arterial cannulation, allowing safe reinfusion in case of excessive bleeding (Figure 1a). The aorta is then tractioned caudally using long arterial forceps to both approximate and stabilize the cannulation site. Before cannulation, the lungs are deflated to minimize physiological movement. After insertion of the HLS Cannulae (Maquet Cardiopulmonary GmbH, Rastatt, Germany), the cannula is securely fixed with two tourniquets, each secured with silk sutures. To achieve optimal exposure of the target vessels, it is imperative that the pulmonary veins and the inferior vena cava be encircled with vascular tape at the onset of cardiopulmonary bypass, thereby preventing major vascular injury.

The Chitwood DeBakey Clamp (Scanlan^®^ International Inc., Minneapolis, MN, USA) is inserted into the thoracic cavity through the anterior axillary line of the second intercostal space, and an aortic cross-clamp is applied. Cardioplegia is administered every 20 min via the MiAR™ antegrade cardioplegia cannula (Medtronic Inc., Minneapolis, MN, USA). The proximal anastomosis is performed with a side clamp placed on the ascending aorta after cross-clamp removal (Figure 1b,c). Sequential anastomosis was frequently preferred when the coronary anatomy was suitable. After decannulation, the pledgetted sutures previously placed in the ascending aorta are then tied off. The procedure is finished by achieving hemostasis.

### 2.4. Clinical Parameters and Outcomes Measures

The primary parameters evaluated in the study are as follows: operative mortality, neurological deficit, conversion to sternotomy during surgery, major bleeding from the aortic cannulation site, and aortic complications (e.g., aortic dissection). Additionally, the study assessed delirium, reoperation for bleeding, surgical site infection, intensive care unit stay, and hospitalization. Operative mortality was defined as death occurring during hospitalization or within 30 days of surgery, regardless of the cause. The term “stroke” was defined as a new-onset transient or permanent neurological deficit, clinically or radiologically evident, occurring in the postoperative period due to hemorrhagic or ischemic causes. Postoperative neurological assessment was performed during daily clinical examinations. Neurological consultation and radiological imaging were reserved for patients presenting with new-onset focal neurological deficits or unexplained changes in consciousness.

### 2.5. Statistics

Statistical analysis was conducted using IBM SPSS Statistics for Windows (version 25.0; IBM Corp., Armonk, NY, USA). Continuous variables were tested for normality with the Shapiro–Wilk test. Normally distributed continuous variables were reported as mean ± standard deviation, while non-normally distributed variables were shown as median (interquartile range). Categorical variables were presented as frequency and percentage. Outcomes were assessed using descriptive statistics. Operative mortality and postoperative complication rates were calculated as percentages.

## 3. Results

During the study period, 138 patients underwent TCRAT at our institution. In 29 cases, the procedure was performed using antegrade arterial perfusion. The baseline characteristics are listed in Table 1.

In all cases, the LITA was used as the primary graft. The great saphenous vein served as an additional graft in this cohort. A detailed analysis of the surgical distribution reveals that an isolated left anterior descending coronary artery bypass graft (CABG) was performed in 2 patients, 2-vessel CABG in 5 patients, 3-vessel CABG in 14 patients, 4-vessel CABG in 5 patients, and 5-vessel CABG in 3 patients. The mean number of distal anastomoses per patient was 3.07 ± 0.9. Complete revascularization was defined anatomically as grafting all coronary arteries > 1.5 mm in diameter with >50% stenosis. Intraoperative variables are presented in Table 2. Additionally, coronary endarterectomy was performed on the right coronary artery in one patient and on the first diagonal artery in another patient. No patient required conversion to sternotomy. No patient experienced cannulation site–related complications, such as significant bleeding or aortic dissection. No vascular, thrombotic, or infectious complications were observed at the percutaneous venous cannulation site during the early postoperative period. No patients required reoperation in the early postoperative period.

One patient died from severe pneumonia on the postoperative ninth day. No perioperative myocardial infarction or stroke was observed. Postoperative delirium was noted in one patient. During hospitalization, atrial fibrillation was observed in two patients. Both cases were restored to sinus rhythm with medical treatment. The average ICU stay was 1.2 ± 0.4 days, and the average hospitalization duration was 5.3 ± 1.1 days. One patient developed a superficial wound infection at the thoracotomy site, which was resolved with medical treatment and conservative management.

## 4. Discussion

Beyond the cosmetic benefits of minimally invasive surgical techniques, numerous studies indicate that these techniques reduce surgical trauma, thereby lowering blood transfusions, shortening hospital stays, and facilitating faster patient recovery and earlier return to daily activities [11,12,13]. Alongside technological advances in percutaneous coronary interventions, less invasive approaches have also been developed in CABG surgery. These include minimally invasive direct coronary artery bypass (MIDCAB), totally endoscopic coronary artery bypass (TECAB), automated distal anastomosis systems, and minimally invasive techniques for multiple vessel revascularization [1,2,14,15].

Minimally invasive off-pump coronary artery bypass surgery offers significant advantages, including substantially reducing the risk of stroke by decreasing aortic manipulation and preserving organ function and hemostasis [16]. A meta-analysis of 37,720 patients reported that decreasing the degree of aortic manipulation significantly lowered stroke risk. Off-pump coronary artery bypass surgery was associated with a 78% lower risk of stroke than conventional on-pump surgery [17]. This translates into lower mortality and morbidity, particularly among high-risk patient groups. However, randomized controlled trials have shown that coronary artery bypass surgery with cardiopulmonary bypass yields higher graft patency rates and lower reintervention rates for revascularization than off-pump CABG [18,19]. Furthermore, surgery on an arrested heart provides the surgeon with a motionless, bloodless surgical field, allowing for more precise, easier anastomoses.

In 2019, the TCRAT technique described by Babliak and colleagues was reported as a safe, sternum-sparing method for total arterial revascularization via left anterior thoracotomy, using cardiopulmonary bypass and cardioplegic arrest [4]. While midterm results reported in the literature are promising, the need for peripheral arterial cannulation presents a significant limitation, especially for patients with peripheral vascular disease [10,11,12,20]. Additionally, the increasing risk of stroke in cases of minimally invasive valve surgery with femoral cannulation raises concerns.

Several studies have reported complications linked to peripheral artery cannulation, including cerebrovascular events, retrograde aortic dissection, vascular trauma, distal ischemia, and surgical site and wound infections [11,21,22]. Gammie et al. reported a 1.96-fold increase in stroke risk in patients undergoing minimally invasive mitral valve surgery compared to median sternotomy, based on the Society of Thoracic Surgeons Adult Cardiac Surgery Database [23]. However, the absence of a clear definition of minimally invasive mitral valve surgery in the database has led to the hypothesis that neurological complications may be mainly associated with femoral artery perfusion rather than the incision site. The subsequent 2011 International Society for Minimally Invasive Cardiothoracic Surgery consensus report, based on a meta-analysis of 35 studies, indicated that the risk of stroke might be higher with the minimally invasive approach [24].

Retrograde perfusion might destabilize or detach previously stable plaques in the femoral, iliac, or aortic regions. The high flow velocities required to achieve the target flow rate through femoral artery cannulation can cause jet flow effects and increased shear stress, particularly in patients with large body surface areas. This may lead to local microtears on the atherosclerotic plaque surface, surface vibration, and possible plaque detachment, even in small, stable plaques. Numerous studies have systematically examined the association between retrograde arterial perfusion and stroke development in minimally invasive cardiac surgery, particularly in mitral valve procedures. In a retrospective analysis of 1282 patients undergoing first-time mitral valve surgery, Grossi et al. reported that retrograde perfusion increased the risk of stroke in cases involving severe peripheral artery disease and other risk factors [25]. Furthermore, another study has demonstrated that retrograde arterial perfusion significantly increases the risk of stroke and is an independent predictor in patients aged seventy years and older with an atherosclerotic process [13].

The prevalence of abdominal aortic atherosclerotic plaques increases with age [26,27]. Also, measurable abdominal aortic calcification has been observed even in individuals without typical cardiovascular risk factors, such as hypertension, dyslipidemia, diabetes, smoking, and obesity, at age 55 [28]. Furthermore, research has shown a correlation between coronary artery disease and the presence of atherosclerotic plaques and calcifications in the descending aorta and peripheral arteries [29,30]. The existing literature suggests a strong correlation between the severity of plaques and calcifications in the descending aorta and the severity of coronary artery lesions, regardless of other risk factors. Therefore, careful planning is essential for femoral artery cannulation in patients scheduled for minimally invasive surgical revascularization. Conversely, studies conducted at high-volume centers (Cleveland, Leipzig, and Mayo/UPenn) have reported no significant differences in stroke risk between retrograde and antegrade perfusion [31,32,33].

In the present study, the mean Body Mass Index of patients was 33.2 ± 5.4 kg/m^2^. Notably, femoral cannulation is not contraindicated in obese patients. However, adipose tissue is highly sensitive to mechanical damage, and given that its blood supply is provided by terminal vessels, even minor damage can lead to necrosis of the entire tissue unit. Fat necrosis prolongs the inflammatory response and delays healing. Moreover, the groin area, which is anatomically prone to sweating and bacterial accumulation, is much more vulnerable to infection in obese patients. Similar factors have been posited to explain the increased risk of wound site infection after cardiac surgery, with obesity found to increase the risk by approximately 29% [34]. Furthermore, surgical dissection in the groin area more frequently causes lymph leakage and lymphocele formation in obese patients. Such wound complications are a significant cause of morbidity that prolongs the hospital stay of obese patients. Furthermore, central arterial cannulation is technically less challenging in obese patients than in other patient groups because of their generally wider intercostal spaces, which is another reason we prefer central arterial cannulation in this patient group.

Sellin et al. concluded that axillary artery cannulation may be useful as a peripheral approach in patients designated for receiving TCRAT [12]. It is undoubtedly an alternative to femoral access, but peripheral arterial plaques or calcifications still have debates. Upper-extremity arteries also have much smaller diameters, which may not be suitable for larger cannulas to achieve sufficient flow rates without elevated pressure. In addition, axillary artery cannulation is more difficult due to its location and its relationship to the clavicle.

Central aortic cannulation is increasingly used in minimally invasive valve surgery at experienced medical centers. There is a widespread perception that central aorta cannulation is challenging, primarily due to the learning curve and the risk of bleeding at the cannulation site. Robot-assisted or direct-view minimally invasive cardiac surgical procedures have been performed routinely at our clinic for approximately 10 years. Our growing experience with minimally invasive mitral valve surgery via right thoracotomy, coupled with literature highlighting the increased risk of stroke and vascular complications associated with peripheral cannulation, has led us to adopt a central aortic cannulation strategy for valve surgery. This study presents our first 29 cases in which we applied central aortic cannulation in the TCRAT procedure. The technical proficiency gained over the past decade in our valve surgery practice has been the most important factor enabling central cannulation in TCRAT cases. The following key lessons have been identified and prioritized to facilitate the process. As previously reported, the TCRAT is performed at the 3rd and 4th intercostal spaces in our clinic [10]. Thoracotomy is routinely performed at the third intercostal space, particularly when central arterial cannulation is planned, to ensure optimal exposure of the aorta. To avoid complications related to the LITA graft, it is crucial not to divide the graft immediately after thoracotomy. Instead, the proximal segment is harvested first, and the distal ITA is carefully harvested to the needed length by reversing the retractor. To prevent restricting the proximal anastomosis site, central arterial cannulation is performed as distally as possible on the ascending aorta. Several technical considerations are essential for safe and controlled cannulation. These include fully opening the pericardium, applying positive end-expiratory pressure to the contralateral lung, carefully rotating and applying mild traction to the aorta, and deflating the lung before cannulation to immobilize the aorta. Additionally, from a safety standpoint, cannulating the femoral vein before arterial cannulation allows rapid reperfusion if needed. It provides a significant safety margin in the event of unexpected hemodynamic complications.

The main advantages of central aortic cannulation include maintaining antegrade, physiological flow; reducing complications associated with peripheral artery cannulation; avoiding an additional surgical incision; and providing a reliable alternative to axillary artery cannulation when femoral cannulation is contraindicated due to peripheral artery disease. Additionally, central cannulation provides improved surgical safety by enabling more predictable hemodynamic control and stable perfusion pressures during cardiopulmonary bypass. Although direct aortic cannulation was performed in this series, the Seldinger technique with a guidewire remains a viable and effective alternative. This approach may offer additional safety and ease of use, particularly in patients with limited surgical exposure or unfavorable anatomical positioning of the ascending aorta.

The current study has some limitations. Its retrospective, non-comparative design, which does not include patient-matched control groups across different time periods and cannulation techniques, limits the interpretation of the results. Additionally, there may be selection bias in patient selection between retrograde and antegrade arterial perfusion. Also, the relatively small number of cases lacks the statistical power to detect rare but catastrophic complications such as stroke or aortic injury. This does not imply that these risks are eliminated, but rather reflects the preliminary nature of our experience with central aortic cannulation in TCRAT. The study also covers only early postoperative results; therefore, mid- and long-term clinical outcomes require further examination. Despite preoperative or intraoperative investigations (CT angiography, iliofemoral ultrasound, TEE), the absence of systematic epiaortic ultrasound prevents complete exclusion of silent embolic events that may occur during direct manipulation of the ascending aorta (cannulation, clamping, and side clamping). Hence, these findings are preliminary and should be confirmed through prospective, randomized, multicenter studies with larger patient populations to either support or challenge the current conclusions.

## 5. Conclusions

Although more technically challenging than femoral artery cannulation, these preliminary findings suggest that central arterial cannulation may be a safe and practical alternative for the TCRAT technique, potentially expanding the use of minimally invasive coronary revascularization. This method, which offers the potential to reduce the risk of neurological and vascular complications associated with retrograde perfusion by providing antegrade, physiological flow, may offer a broader application for the use of the TCRAT technique, especially in patients with peripheral artery disease, advanced descending aortic atherosclerosis, or those unsuitable for femoral access. However, the results reported here are based on preliminary data from an early-phase study and involve a small number of patients. Therefore, larger, prospective, randomized multicenter studies are needed to conclusively determine the safety and effectiveness of central aortic cannulation in TCRAT and to either support or challenge these initial findings.

## Figures and Tables

**Table 1:** Baseline Characteristics of the Patients.

Variable	Value
Age (years)	57.2 ± 9.8
Sex	
Male, ***n*** (%)	21 (72.4%)
Female, ***n*** (%)	8 (27.6%)
Active Smoking, ***n*** (%)	22 (75.9%)
Diabetes Mellitus, ***n*** (%)	15 (51.7%)
Prior percutaneous coronary intervention, ***n*** (%)	4 (13.7%)
Hypertension, ***n*** (%)	18 (62.1%)
Previous Stroke, ***n*** (%)	0 (0%)
Peripheral Artery Disease, ***n*** (%)	8 (27.5%)
Preoperative Ejection Fraction (% ± SD)	57.6 ± 6.6
Preoperative EuroSCORE II (median)	2.0% (IQR: 1.2–3.8)
Body Mass Index (kg/m^2^ ± SD)	33.2 ± 5.4

**Table 2:** Intraoperative variables.

Target Coronary Artery	*n* (%)
Left Anterior Descending Artery	29 (100%)
First Diagonal Branch	14 (48.2%)
Second Diagonal Branch	4 (13.7%)
Intermediate artery	3 (10.3%)
Obtuse Marginal Branch	18 (62%)
Right Coronary Artery (RCA Main/Trunk)	10 (34.4%)
Posterior Descending Artery	6 (20.6%)
Cardiopulmonary Bypass Time (minutes)	118.6 ± 41.6
Cross-Clamp Time (minutes)	63.2 ± 27.0
Sequential anastomosis	18 (62.1%)

## Data Availability

The data from this study are available upon request from the corresponding author.

## Abbreviations

The following abbreviations are used in this manuscript:

CT	Computed Tomography
CABG	Coronary Artery Bypass Graft
LITA	Left Internal Thoracic Artery
MIDCAB	Minimally invasive direct coronary artery bypass
TCRAT	Total coronary revascularization via left anterior thoracotomy
TECAB	Totally endoscopic coronary artery bypass
TEE	Transoesophageal Echocardiography
